# Green space attributes and their impact on perceived stress in Poland

**DOI:** 10.1038/s41598-025-98707-4

**Published:** 2025-04-22

**Authors:** Dagmara Stangierska-Mazurkiewicz, Beata Fornal-Pieniak, Paweł Szumigała, Katarzyna Widera, Barbara Żarska, Karolina Szumigała

**Affiliations:** 1 Department of Pomology and Horticulture Economics, Institute of Horticultural Sciences, Warsaw University of Life Sciences—SGGW, Nowoursynowska 166, 02-787 Warsaw, Poland; 2 Department of Environmental Protection and Dendrology, Institute of Horticultural Sciences, Warsaw University of Life Sciences—SGGW, Nowoursynowska 166, 02-787 Warsaw, Poland; 3 Department of Landscape Architecture, Faculty of Agronomy, Horticulture and Bioengineering, Poznań University of Life Sciences, ul. Dąbrowskiego 159, 60-594 Poznan, Poland; 4 Department of Economics, Finance, Regional Research and Quantitative Methods, Faculty of Economics and Management, Opole University of Technology, Prószkowska 76, 45-758 Opole, Poland; 5 Department of Civil Engineering and Geoengineering, Faculty of Environmental and Mechanical Engineering, Poznań University of Life Sciences, ul. Piątkowska 94, 60-649 Poznan, Poland

**Keywords:** Urban greenery, Green space, Perceived stress, Perception and experience, Environmental perception, Psychology and behaviour, Human behaviour

## Abstract

Inconsistent findings and limited research from various countries highlight the need for further investigation of the relationship between Satisfaction with Green Space Attributes (SGSA) and Perceived Stress (PS) levels, which is crucial for informing urban planning strategists to improve residents’ mental health using green areas. Presented study explored these relationships and differences in greenery usage and preferences in connection with self-perceived stress levels in the study area of post-socialist cities and other settlements units in Poland—the country belonging to the former block of Socialist Countries of People’s Democracy (specific type of urbanism and influence on mentality of residents). Data collected in 2022 via Computer-Assisted Web Interviewing (CAWI) covered demographics, green space utilization, SGSA, and PS levels using the Perceived Stress Scale (PSS-10). Statistical analyses, including Kruskal–Wallis rank ANOVA, Mann–Whitney U-test and multiple regression, revealed that lower PS levels were correlated with higher SGSA across various greenery elements. Individuals with low stress levels spent more time in green spaces for leisure activities, mainly walking. However, satisfaction with greenery decreased with increasing stress levels, especially regarding the decrease of cleanliness, aesthetics and greenery amount. Multiple regression identified significant predictors of stress levels, such as health, greenery aesthetics satisfaction, income, and green area accessibility for children. The study underscores the importance of well-designed, diversified green infrastructure to enhance residents’ mental well-being. Accessible, well-maintained green spaces creating a network within urban environments are crucial for stress reduction. It is a guideline for policymakers and urban planners to create continuous green infrastructure consisting of various size and character green areas/elements and this green network should be as dense as possible, occupying every possible place.

## Introduction

The modern urban lifestyle of city residents is shaped by various socio-economic factors and the ongoing process of urbanization, which significantly contributes to increasing stress levels among individuals^1,2^. Urban green spaces are commonly considered for appreciating scenic beauty, relaxation, and psychological restoration following mental fatigue^3^. The capacity of nature to reduce stress in humans evokes positive affective states and enhances cognitive functioning, what is paramount for residents of large cities. Grahn and Stigsdotter^4^ confirmed long time ago the connection between the use of urban open green areas and self-reported experiences of stress, but regardless of respondents’ age, sex, and socio-economic status, among residents of Swedish cities; the great significance of the green areas available in everyday life was emphasized. What is often foregrounded as empirical evidence supporting the beneficial effects of urban greenery (green infrastructure) on mental health is the wealth of decades-long research in environmental psychology, including ecological behaviors. Such research has confirmed the potential for psychological restoration through exposure to vegetation and other natural elements^5,6^. Urban green spaces, enriched with various trees, water features and diverse vegetation, garner interest among people and aid in psychological stress recovery through their utilization. Therefore, it is recommended that green areas of varying sizes should be designed to appeal not only visually by incorporating diverse compositions of plants, flower colors and foliage^7^. Kaplan and Talbot^8^ introduced the term „restorative environments” 40 years ago, providing numerous benefits to humans (residents of cities), including reduction of stress. These “restorative environments” (green areas in cities: remnants of wild nature and greenery purposefully designed and implemented by humans) are vital because of such critical factors involved with them: (1) *being away* (away from the everyday environment), (2) *fascination* (or interest) (users not to be bored), (3) *coherence* (functioning as a “another world” for user), (4) *compatibility* (wilderness calls on users’ psychological predispositions/inclinations being shaped during long course of evolution). Also, many research from years 2010–2023 confirm beneficial associations between green spaces and human health^9^. There is growing scientific evidence that nature in general, and wilderness in particular, make a substantial difference in the various benefits residents gain from using green spaces^7,10^. For example, benefits such as contemplation and self-insight, especially during the COVID-19 lockdown, are more likely to be better provided by the natural environment, particularly by wilderness^11^.

It has been observed that accessibility, adequate infrastructure, maintenance status, and proximity of green spaces for urban residents emerged as critical criteria assessed by respondents to verify the benefits of green areas for human health. It has also been noted that for urban populations to deliver maximum health benefits from green infrastructure, greater attention should be paid to accessibility, quantity, quality, location of green spaces, and their management^10,12^.

The composition of plantings is also crucial. When selecting species of perennials and grasses, particular attention should be paid to their seasonal variability, focusing on plant structure. For designers, the form of the plant before, during, and after flowering, as well as leaf shape, are important considerations. All these plant characteristics are highly significant in horticultural therapy, which involves "healing through plants, via scent, color, and aesthetic qualities." As the primary component of green spaces (urban greenery), plants significantly impact residents’ regeneration after psychological stress, as confirmed by numerous scientific studies^7^. Interactions between people and green spaces improve mental well-being, enhance mood and attention, and reduce stress and anxiety^13^. These previous research concentrate rather on “construction” of green areas to deliver a variety of stimuli for user’s senses, than finding multi-faceted specificity of correlation between using of green areas and regulating self-perceived stress level which conditions directly self-perceived well-being.

Furthermore, the Covid-19 pandemic in the years 2020–2021 had a tremendous negative impact on people’s mental and physical health. During the pandemic, green spaces were identified as the most valuable and safest places for gatherings with family and friends to reduce stress induced by the prevailing situation^11^. Perceiving the benefits/merits of green spaces and spending time surrounded by vegetation positively influence psychological rejuvenation^14,15^.

Urban greenery provides broad health benefits, including the reduction of stress and the risk of psychiatric disorders^16^. Natural environments are more effective in lowering stress than urban spaces^17^, and contact with nature reduces anxiety^18^. Urban greenery reduces stress and improves well-being^19^, and a greater amount of green space helps mitigate the effects of stressful events^20^.

Beil and Hanes^21^ observed a significant improvement in post-exposure stress measurements reported by participants (especially women) exposed to urban natural environments compared to built-up environments. In biodiversity conservation and improving human well-being, a comprehensive approach to environmental issues related to preserving existing green spaces and designing new green areas is essential in European cities^22^. These prior studies consider indeed the relation between green areas and stress level, but examine rather general aspects, not regarding deeper to more detailed characteristics.

The inconsistent findings from previous studies and the need for studies from various countries (of diverse types of development, different cultures, different regions of the World) necessitate more research, especially in the field of relationship between using green areas and self-perceived stress level and well-being. Hence, the primary aim of this study was to investigate the connections between Satisfaction with Green Space Attributes (SGSA) and Perceived Stress (PS) levels. The secondary objective of this study was to explore deeper the differences between the usage and preferences of greenery in relation to varying stress levels. The study area is specific: the post-socialist cities and other settlement units in Poland belonging to the former block of Socialist Countries of People’s Democracy. The period 1945–1989 of socialist development in this area has exerted significant influence on different type of urbanism and on specificity of residents’ mentality. There are significant differences in development between cities in western and eastern Europe after the World War II. The detailed objectives of the study were as follows:

1. Determining residents’ preferences regarding using urban greenery concerning reduction of stress levels.
2. Identifying how satisfaction with specific urban greenery elements corresponds with stress levels.
3. Obtaining a quantitative understanding of the impact of various factors related to urban greenery and respondent profiles on stress levels using multiple regression analysis.
4. Providing recommendations for urban green space planning to restore better the mental health of residents.

## Methods

### Study design and data collection

The study utilized data gathered from a questionnaire administered in 2022 through the Computer-Assisted Web Interviewing (CAWI) method, facilitated by Google Forms. Before initiating the questionnaire, participants received a concise overview of the study’s objectives and were assured of anonymity and confidentiality. Participants remained anonymous throughout the survey, as they did not provide their names or identifiable information, including IP addresses. The study did not involve the collection of sensitive personal data, health-related information, or any experimental procedures. Additionally, it adheres to the General Data Protection Regulation (GDPR 679/2016), with measures such as data pseudonymization and aggregation further safeguarding participant privacy. They retained the autonomy to discontinue the study at any stage, with their responses omitted. The online survey complies with national and international regulations, aligning with the Declaration of Helsinki (2000). Furthermore, the study adhered to ethical guidelines for social science research, ensuring that participants faced no foreseeable risks or harm.The European Parliament’s General Data Protection Regulation (GDPR 679/2016) anonymized participants’ details to safeguard their privacy. Written informed consent was obtained from all participants before they proceeded with the survey. Participation was voluntary, and individuals were recruited through personal contacts and social media channels. Overall, the survey encompassed a diverse group of participants; however, there was a notable overrepresentation of young, educated women from urban areas, likely due to the voluntary nature of participation and the online CAWI methodology.

The recruitment of participants primarily utilized social media, particularly Facebook and specialized groups focused on green spaces and quality of life. A post containing the survey link was shared on Facebook and within these groups, along with a request for respondents to complete the survey and share it with their acquaintances. Additionally, the survey was distributed via email, with similar instructions encouraging others to participate.

Some respondents agreed to promote the survey link within their networks and social organizations. Participants were informed that they could also share the link through email, Facebook, or other channels.

No paid advertisements or compensation of any kind were offered for completing, sharing, or engaging with the survey. While the survey link was occasionally promoted through social media posts and advertisements, including a dedicated Facebook study page, it was not incentivized in any way. As a result, it is not possible to accurately estimate the percentage of respondents who participated through the snowball method or personal contacts, such as distributing the survey among friends, family, and colleagues. The online recruitment approach led to an overrepresentation of younger individuals.

This study was conducted in accordance with Polish regulations, specifically the Act of 5 December 1996 on the Professions of Physician and Dentist (Ustawa z dnia 5 grudnia 1996 r. o zawodach lekarza i lekarza dentysty, with amendments), which does not require ethical committee approval for survey-based research that does not involve medical interventions, invasive procedures, or the collection of sensitive data. Since the study only involved anonymous self-reported survey responses with no risk to participants, obtaining ethical approval was not required under national and institutional regulations. The study complied with all relevant ethical standards and adhered to the principles outlined in the Declaration of Helsinki.

### Questionnaire

The questionnaire related to urban green space standards and the level of satisfaction derived from everyday life. The research questionnaire consisted of three main parts. The first part of the questionnaire contained questions about the characteristics of the respondents’ taking into account gender, age, place of residence, education, income level and health declaration.

Questions of the second part of the questionnaire asked about two main aspects to determine the preferences regarding the use and satisfaction with green spaces. First, we ask questions about spending free time on green spaces (frequency question with answers: daily, 5–6 times a week, 3–4 times a week, 1–2 times a week, less than once a week). Then was a section of more detailed questions regarding the frequency of green space use by different activities in green areas by respondents, which was scored from 1 to 5, where one signifies "never," 2—"almost never," 3—"sometimes," 4—"quite often," and 5—“very often”. Green space activities for which respondents indicated the frequency are: walking, leisure, exercise and sports, being outdoors, spending time with family and friends, exposure to nature, and health reasons. Questions about satisfaction with green space attributes (SGSA) in the place of residence were developed based on modifications and expansions of tools utilized in other studies^23–25^. Selected from the review of tools and independently developed questions were measured on a 5-point Likert scale from 1—totally disagree to 5—totally agree concerned on essential sites. The final list comprised 16 attributes of satisfaction with urban green spaces:

- Overall satisfaction with the place of residence
- Quantity of greenery
- State of cleanliness and aesthetics
- Proximity of large parks
- Distance from the forest
- Availability of green areas where children can play.
- Proximity of green areas where you can sit and relax
- Availability of green areas where you can practice sports
- Proximity of green areas where you can walk your dog
- Presence of green belts between the sidewalk and the street
- Presence of small ornamental trees along the streets
- Presence of large, spreading trees along the streets
- Footpaths
- Night lighting
- The number of water elements in green areas
- The aesthetics and functionality of water features.

The questionnaire, in the third part, incorporated the Perceived Stress Scale (PSS) to assess the primary outcome variable—stress. This is a widely used tool developed by Cohen, Kamarck, and Mermelstein, consisting of ten items with Likert-scale responses, resulting in scores ranging from 0 to 40^26^. Higher scores indicate higher levels of perceived stress.

In this study, the Polish adaptation of the PSS-10, developed by Juczyński and Ogińska-Bulik, was used. Its psychometric validity and reliability have been confirmed in numerous studies^27^. The Polish version demonstrated a high internal consistency coefficient (Cronbach’s alpha = 0.86), and test–retest reliability was 0.90 over a two-day interval and 0.72 after four weeks. Convergent and discriminant validity of the scale was confirmed through correlations of PSS-10 scores with levels of depression, anxiety, and mental health^28^.

Raw PSS-10 scores were converted into a sten scale according to norms established for the Polish population^27^. Subsequently, the raw score is converted into a sten scale. Scores within the range of 1–4 are considered low stress, stens 5 and 6 as moderate, and 7–10 as high levels of perceived stress. Moreover, its suitability for self-administration in surveys is noteworthy, and previous studies, such as one examining stress and green space by Ward Thompson et al.^29^, have utilized it, ensuring comparability. For statistical analyses, the three stress levels (low, moderate, and high) were used for nonparametric tests, while raw PSS-10 scores were applied in multiple regression analyses..

### Basic information about the participants

Regarding gender, the sample consisted of 65% women and 35% men. The age structure of the studied group was: 18–25 years old—53%; 26–35 years old—17%; 36–55 years old—22%; 55 and older—8%. In terms of education, over half of the respondents were people with higher education (52.75%); 42% of the respondents had completed secondary education, and less than 7% had a lower-than-secondary level of education. Nearly 50% of the respondents lived in cities with a population larger than 200,000 inhabitants; the second biggest group was rural area residents, almost 24%. Despite the questionnaire focusing on urban green space, respondents include individuals residing in rural areas. However, this stems from the current lifestyle dynamics where rural areas serve as places of residence while work and most daily necessities are carried out in urban areas. Nearly 15% lived in towns with under 50,000 people and 13% in cities with 50–200 thousand residents. Regarding income demographics, the distribution within the sample was diverse. Most respondents fell into the middle-income brackets, with almost 24% reporting incomes ranging from PLN 2501-3500, followed closely by 21% within the PLN 1501-2500 range. Subsequent income brackets included over 16% earning between PLN 3501-4500, 15.75% reporting incomes over PLN 5500, 11% earning under PLN 1500, and an equal proportion of 11% falling within the income range of PLN 4501-5500. The most significant portion of the sample comprised those with moderate stress levels (nearly 60%). The low and high-stress groups accounted for 19.5% and 20.8%, respectively (Table 1).

**Table 1:** Descriptive characteristics of the sample (N = 692).

Characteristic	n (%)
Gender	
Female	452 (65.32%)
Male	240 (34.68%)
Age	
18–25	365 (52.74%)
26–35	116 (16.76%)
36–55	152 (21.97%)
Over 55 years old	59 (8.53%)
Education	
Primary and Vocational	46 (6.65%)
Secondary	291 (42.05%)
Higher	355 (51.30%)
Residence	
Village	164 (23.70%)
A city with up to 50 thousand inhabitants	103 (14.88%)
A city with 50 to 200 thousand inhabitants	90 (13.01%)
A city with more than 200,000 inhabitants	335 (48.41%)
Income per capita PLN	
Under 1500	79 (11.42%)
1501–2500	147 (21.24%)
2501–3500	164 (23.70%)
3501–4500	114 (16.47%)
4501–5500	79 (11.42%)
Over 5500	109 (15.75%)
Level of perceived stress (PSS-10)	
Low	135 (19.51%)
Moderate	413 (59.68%)
High	144 (20.81%)

### Statistical methods and data analysis

Typical data cleaning procedures were conducted, involving the review of responses for potential errors and screening for incomplete or non-responses. For instance, the survey included a question on income. Still, because this question often discourages respondents from participating in the study, it needed to be more moderate. For analysis, only surveys where respondents selected an income range were considered, necessitating the removal of 36 surveys during the initial processing stage.

Variables were identified from data collected in an empirical study to verify research hypotheses. The variables in the study were questions to the respondents, and their answers constituted the empirical material that formed the basis for this verification. The measurement scale on which these variables were measured is the rank and nominal scale. Despite the large size of the research sample, statistical tools adequate to these scales of measurement were used. Therefore, non-parametric tests were used, which are adequate for the analysis of variables measured on qualitative scales. To verify the hypotheses, the statistical analysis assumed a significance level of α = 0.05, and a side effect of η^2^ was calculated. A Kruskal–Wallis rank ANOVA study was conducted for more than two independent groups to assess whether stress levels affect leisure time spent in green spaces. The nonparametric Mann–Whitney U test, used in pairs for two independent groups, facilitated the determination of statistical significance or lack thereof in preferences related to use and satisfaction with greenery among different stress groups (PSS-10).

An advanced tool for statistical data analysis, i.e. the multiple regression model, was also used, the purpose of which was to describe and explain the formation of the explanatory variable—the PSS-10 level. The PSS-10 scale results, categorized into three stress levels (low, moderate, high), were used in nonparametric tests. However, for multiple regression analyses, raw PSS-10 scores were applied to ensure a more precise assessment of relationships. In the process of developing the model, both the variables from the main part of the survey and demographic variables (age, gender, education, urban/rural residence) were tested for their potential influence on the relationships under investigation. However, the demographic variables did not reach statistical significance (p > 0.05) and were therefore excluded from the final model. This suggests that demographic factors did not substantially affect the observed relationships. The structural parameters of the econometric regression model were estimated until their statistical significance was obtained (at the assumed level). This allowed to obtain a model equation in which all identified explanatory variables significantly indicate a cause-and-effect relationship between the perceived stress level (PSS-10) and the preference for greenery.

The properties of the obtained model were also verified by examining the correspondence of the distribution of residues with the normal distribution. A nonparametric χ^2 Pearson test of independence was used, the value of the test statistic confirmed the achievement of this agreement. All calculations were performed using Statistica 14.1.

### Ethical statement

All subjects gave informed consent for inclusion before participating in the study. The study was conducted following the Declaration of Helsinki.

## Results

### Use of green spaces and stress

People with the lowest levels of perceived stress (LPS) report spending their leisure time in green areas most often every day, compared to those with higher levels of perceived stress. Among the three analyzed groups, those with the lowest levels of perceived stress also reported the highest total percentage of spending their free time in green spaces at least 5 times a week. In contrast, those with the highest levels of perceived stress reported this frequency the least often. Individuals experiencing moderate levels of perceived stress tend to spend their leisure time in green spaces approximately 3 to 4 times a week. In contrast, those with the highest levels of perceived stress typically visit these areas only 1 to 2 times a week. Consequently, individuals with the highest perceived stress reported the lowest frequency of spending their leisure time in green spaces. A Kruskal–Wallis rank ANOVA test revealed significant differences in the time spent in green spaces based on perceived stress levels, although the effect size was small. The study involved 692 diverse participants, including many younger respondents, whose preferences may have influenced the results. While these differences are statistically significant, their practical implications are limited. (Table 2).

**Table 2:** The overall frequency of spending leisure time in green spaces due to the level of perceived stress (Kruskal–Wallis rank ANOVA).

Frequency of green space utilization	Low LPS	Moderate LPS	High LPS
Daily	23.70%	8.96%	10.41%
5–6 times a week	10.37%	20.34%	10.41%
3–4 times a week	21.48%	32.20%	22.22%
1–2 times a week	27.41%	22.52%	38.89%
Less than once a week	17.04%	15.98%	18.05%

Test Kruskala-Wallisa: H (2, N = 692) = 6.039775, η^2^ = 0.01, p value < 0.05.

Walking was the most common activity in green spaces regardless of the stress level. Individuals with the highest stress levels reported an average frequency of 4.10, while those with the lowest reported nearly 3.8. In second and third place in terms of frequency were the opportunities for relaxation and being outdoors. Exercising and health were the two least significant reasons for spending time in green spaces. However, individuals with the lowest stress levels reported exercising outdoors more frequently than the other two groups. Individuals with moderate and high-stress levels reported a higher frequency of outdoor activity in all analyzed outdoor activities.

**Fig. 1:**
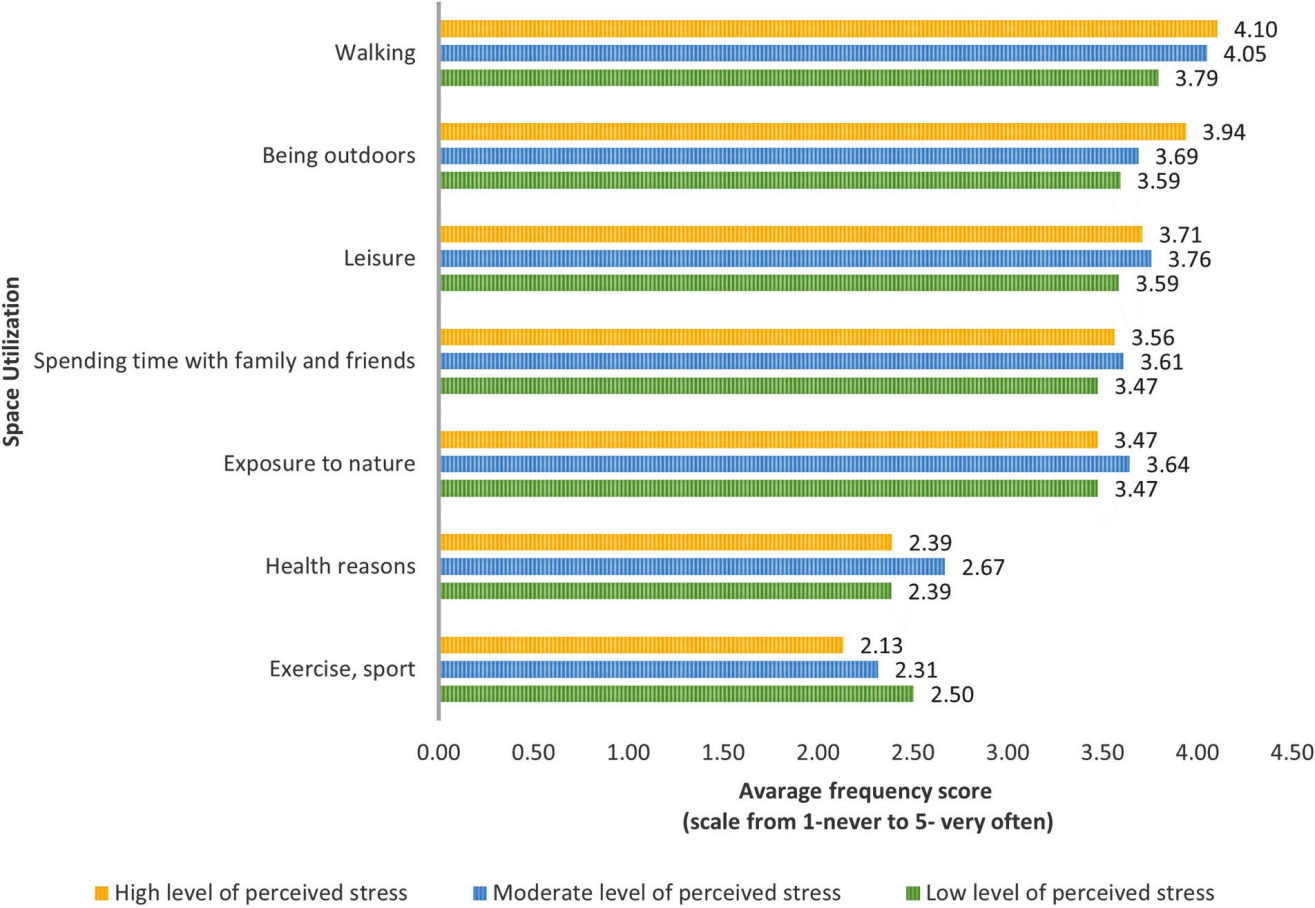
Frequency of Green Space Use comparison of people with different levels of perceived stress (Means).

The ability to relax and be outdoors was ranked second and third independently of the frequency of this activity. The two least important reasons for spending time outdoors were sports, exercise and health, but those with the lowest stress levels were more likely to report exercising outdoors than the other two groups. For all outdoor activities analyzed, those with moderate and high-stress levels reported higher frequencies of outdoor activities (Fig. 1).

The most statistically significant differences, with p-values less than 0.05, were observed in pairwise comparisons using the Mann–Whitney U test among individuals with varying levels of LPS. Notably, individuals with low-stress levels exhibited a significantly lower tendency to report spending time outdoors for leisurely walks and general outdoor activities than those with high-stress levels. Conversely, individuals with low stress levels were significantly more inclined to indicate outdoor activities for exercise. This distinction remained significant when contrasted with individuals experiencing both high and moderate stress levels. Additionally, individuals with high-stress levels were notably more prone to spending time in green spaces for walking when compared to those with moderate stress levels. Furthermore, individuals with moderate stress levels demonstrated a significantly higher likelihood of engaging in outdoor activities for health-related reasons, a trend observed compared to lower and higher stress level groups. Despite the statistical significance of the findings, the relatively small effect sizes may constrain their practical applicability in real-world contexts (Table 3).

**Table 3:** Frequency of Green Space Utilization comparison of people with different levels of perceived stress (pairwise comparisons using the Mann–Whitney U -value Z-test) p-value < 0.05.

Frequency of green space utilization	Low LPS vs moderate LPS	Low LPS vs high LPS	Moderate LPS vs high LPS
Walking	− 2.243*	− 2.183*	− 0.409
Leisure	− 1.228	− 0.611	0.542
Exercise, sport	1.142	2.029*	1.440
Being outdoors	− 0.664	− 2.196*	− 2.064*
Spending time with family and friends	− 0.956	− 0.391	0.590
Exposure to nature	− 1.180	0.109	1.348
Health reasons	− 2.297*	− 0.259	2.169*

*η^2^ = 0.01, p value < 0.05.

### Satisfaction with urban green attributes and stress level

Analysis of overall satisfaction with the place of residence: individuals with low stress levels have an average satisfaction score of 3.59, while individuals with moderate stress levels have an average satisfaction score of 3.38. A similar trend, where an increase in stress levels is associated with a decrease in satisfaction with green infrastructure, applies to 9 of the 15 characteristics surveyed. These features include satisfaction with the amount of green space, cleanliness and aestetics, distance to large parks, paths, lighting, water features, and their aesthetics, as well as ornamental trees along streets. In addition, the availability of seating and relaxation areas was most important for those with the lowest stress levels but the least important for those with moderate stress levels. In the case of the remaining five characteristics, related to the accessibility of green spaces for sports, dog walking, playgrounds, and the presence of grass verges and large trees along streets, people with moderate stress levels showed the highest satisfaction. No such trend was observed.

Individuals with lower stress levels generally reported higher levels of agreement or satisfaction in various areas compared to those with higher stress levels. The most notable differences were observed concerning water elements, particularly regarding their aesthetics and functionality. As stress levels increased, the scores for these elements declined significantly, indicating that individuals with higher stress levels viewed these elements less favorably.

**Fig. 2:**
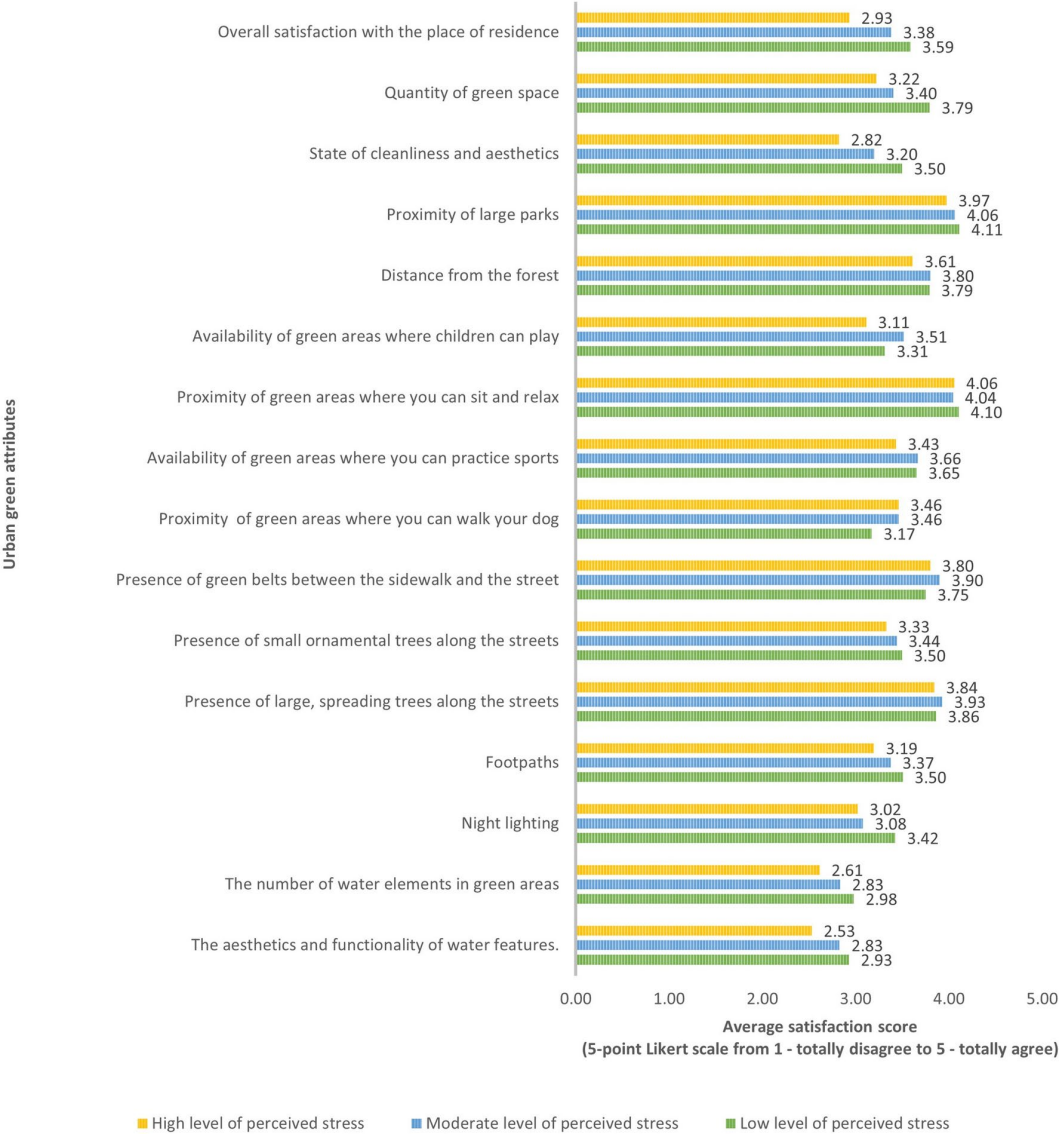
Satisfaction with urban green attributes comparison of people with different levels of perceived stress (Means).

Of all the green elements assessed, people with low and moderate stress levels reported the highest satisfaction with proximity to parks (4.10 and 4.06, respectively). In contrast, people with high stress levels reported satisfaction with the availability of seating and relaxation areas (4.06) (Fig. 2).

The results of the Mann–Whitney U-test showed statistically significant differences between people with low and moderate stress levels in terms of satisfaction with three green space characteristics. The lower the stress level, the more satisfied residents were with the amount of greenery, night lighting, cleanliness, and aesthetics of the greenery. The Mann–Whitney U test revealed significant statistical differences (p-value < 0.05) between individuals with low and high stress levels for 7 out of 16 characteristics. Those with low-stress levels reported significantly higher satisfaction regarding their overall contentment with their place of residence, the amount of greenery, the cleanliness and aesthetics of their surroundings, footpaths, night lighting, the number of water features in green areas, and the aesthetics and functionality of these water features. In comparison, individuals with moderate stress levels expressed greater satisfaction than those with high stress levels, particularly in terms of overall satisfaction with their living environment, cleanliness and aesthetics, availability of green spaces for sports and children’s play, the number of water features in green spaces, and the aesthetics and functionality of these water features. Despite the statistical significance of the findings, the relatively small effect sizes may constrain their practical applicability in real-world contexts. However, it is still important as it provides valuable insights into preferences for green space attributes in connection with levels of stress. (Table 4).

**Table 4:** Satisfaction with Green Space Attributes comparison of people with different levels of perceived stress (pairwise comparisons using the Mann–Whitney U-value Z-test) p-value < 0.05.

Satisfaction with green space attributes	Low LPS vs moderate LPS	Low LPS vs high LPS	Moderate vs high LPS
Overall satisfaction with the place of residence	1.799	4.542*	3.830*
Quantity of greenery	3.450*	3.866*	1.524
State of cleanliness and aesthetics	2.878*	4.859*	3.333*
Proximity of large parks	− 0.055	0.988	1.259
Distance from the forest	− 0.100	1.205	1.615
Availability of green areas where children can play	− 1.227	1.262	3.012*
Proximity of green areas where you can sit and relax	0.539	0.658	0.233
Availability of green areas where you can practice sports	− 0.114	1.618	2.215*
Proximity of green areas where you can walk your dog	− 1.863	− 1.505	0.065
The presence of green belts between the sidewalk and the street	− 1.464	− 0.235	1.304
The presence of small ornamental trees along the streets	0.335	1.177	1.04
The presence of large, spreading trees along the streets	− 0.381	0.287	0.768
Footpaths	1.425	2.299*	1.497
Night lighting	3.148*	3.028*	0.558
The number of water elements in green areas	1.226	2.466*	2.019*
The aesthetics and functionality of water features	0.906	2.934*	2.747*

* η^2^ = 0.01, p value < 0.05.

### The influence of respondent profile and attitudes towards urban green spaces on changes in stress levels

The influence of different urban greening factors and respondent profiles on stress levels was explored using multiple regression, where raw PSS-10 scores were used as the dependent variable. The multiple regression model describing the relationship between the level of perceived stress (YLPS) and preference variables (X1–X7) related to respondents’ characteristics, green space utilization, and satisfaction with greenery (described in Table 5) is given by Eq. (1).

**Table 5:** Parameter values of the multiple regression model describing the relationship between the level of perceived stress and greenery preference and the corresponding p-values.

Variable		Evaluation b	P-value
Health Declaration	X_1_	− 3.236	0.000
State of cleanliness and aesthetics greenery	X_2_	− 0.851	0.001
Being outdoors	X_3_	0.787	0.000
Income	X_4_	− 0.577	0.001
Quantity of greenery	X_5_	− 0.478	0.048
The closeness of green areas where you can walk your dog	X_6_	0.483	0.011
Availability of green areas where children can play	X_7_	− 0.426	0.034
Constant		30.103	

R^2^ = 0.167 p < 0.0000.

1 $$\begin{aligned}{Y}_{LPS} & =30.103-3.236\cdot {X}_{1}-0.851\cdot {X}_{2} \\ & \quad +0.787\cdot {X}_{3}-0.577\cdot {X}_{4}-0.478\cdot {X}_{5} \\ & \quad +0.483\cdot {X}_{6}-0.426\cdot {X}_{7}+e\end{aligned}$$

All structural parameters standing next to the explanatory variables Xi in the model given by Eq. (1) are statistically significant, as all p-values in the table are less than the significance level α = 0.05 assumed in the study.

The interpretation of the evaluation b involves demonstrating how the variable X_i_ influences the change in the value of Y_LPS_—assuming that other variables in the model remain unchanged. This means that a positive value of the evaluation b indicates an increase in Y_LPS_, while a negative value indicates a decrease Y_LPS_, given a unit change in X_i_. To facilitate interpretation, this change has been expressed in terms of unit changes in the PSS-10 score, rather than percentage differences, to ensure clarity and accuracy.

The findings from multiple regression show an improvement in individuals’ health declaration (X_1_) by one unit corresponds to a decrease in the PSS-10 score by 3.236 points, assuming all other variables remain constant. Similarly, a one-unit increase in satisfaction with the cleanliness and aesthetics of green spaces (X_2_) corresponds to a decrease in the PSS-10 score by 0.851 points. As income (X_4_) rises (transitioning from a lower to a higher income group), stress levels decrease by 0.577 points. Moreover, a one-unit increase in satisfaction with the amount of greenery (X_5_) results in a 0.478-point decrease in stress levels. Likewise, a one-unit increase in satisfaction with the accessibility of green areas for children (X_7_) corresponds to a 0.426-point decrease in stress levels (Table 5).

Conversely, stress levels increase by 0.787 points with each unit increase in the frequency of using green areas for outdoor activities (X_3_). Similarly, a one-unit increase in satisfaction with the proximity of the green regions suitable for walking dogs (X_6_) leads to a 0.483-point increase in stress levels.

The proposed model exhibited favorable verification properties, as the analysis of the distribution of its residuals suggests their consistency with a normal distribution. This is corroborated by Pearson’s χ^2^ statistic value of 9.8949 and the corresponding p-value of 0.3591, more significant than 0.05. Additionally, while the model identifies statistically significant relationships, its explanatory power remains limited, as indicated by an R^2^ value of 0.167. This suggests that other factors influencing stress levels were not captured by the included predictors, highlighting the need for further research to account for additional variables.

## Discussion

Our research results indicate that stress levels influence the time spent in green spaces and vice versa. Individuals with low perceived stress (LPS) spend more leisure time in green areas daily than those with higher stress levels. The relationship between stress levels and the frequency of time spent in green spaces was investigated by Van den Berg et al. 2016^30^ in a study conducted in four European cities (Barcelona, Doetinchem, Kaunas, and Stoke-on-Trent). The findings suggest that more time spent in green spaces is associated with higher mental health and vitality scale scores. Research conducted in Singapore by Park et al.^31^ confirms these results, revealing that participants who visited parks during the day reported lower stress levels that evening compared to those who did not visit parks. Furthermore, their study shows that both daily park use and leisure-time physical activity were associated with reduced evening stress. Importantly, these relationships were independent of one another, suggesting that each activity alone contributes positively to lowering stress among adults in urban environments. Zhang et al.^32^ additionally found that heightened exposure to green spaces and diverse green infrastructure was associated with improved physical and mental health outcomes among individuals with hypertension. In alternative studies, rather than exploring the correlation between self-reported time spent in green spaces and stress levels, researchers examined the impact of green space distance and type on stress levels. The findings of the study by Vos et al.^33^ demonstrated that a higher proportion of greenery within a radius of 300 m and 500 m around residential areas was associated with increased likelihood of being more resilient to stress or fear during the COVID-19 pandemic. The study conducted in Denmark by Nielsen and Hansen^34^ revealed that a short distance to green spaces from residential areas and frequent use of green areas and gardens are associated with lower levels of psychological stress. On the other hand, research conducted in Australia demonstrated that exposure to green spaces rich in tree canopy coverage (30% or more) reduces the likelihood of experiencing psychological distress^35^. Also, studies with the help of photostimulation^36^ confirm that there is a positive and concave relationship between the coverage of green areas with perceived happiness. Users declared the highest level of perceived happiness when the green area was covered with trees in 35% and 45%. In contrast, exposure to grass-covered areas showed an increased chance of prevalent psychological distress.

Our study found that walking is the most common activity undertaken in green spaces regardless of the stress level. The high significance of walking as an outdoor activity may be attributed to several reasons. Firstly, respondents included in their responses that they walk in green spaces for pedestrian travel instead of opting for other modes of transportation; this aligns with the findings of research by Wasfi et al.^37^ and Gascon et al.^38^. The second reason for the popularity of walking in green spaces is their visual attractiveness and the elements they contain, which is confirmed by research^39^. A study by Vich et al.^40^ showed that in Mediterranean cities like Barcelona, greenery, including beaches and tree-lined streets, promotes higher physical activity. Meanwhile, research in Greater London demonstrated that park configuration and proximity to retail areas influence the number of walking trips^41^. Additionally, a study by Pinto et al.^42^ found that parks with diverse facilities attract more users across different seasons. Finally, research by Jia et al.^43^ highlighted how the spatial layout of green spaces and their accessibility influence daily walking, also considering urban development needs. Research suggests that to prolong the duration of walking in green spaces, their design should consider the size of the area—appropriately large green spaces incorporating diverse elements, including water features, should be considered. Research findings by Zhang et al.^41^ conducted in London explain the high popularity of walking in green areas by highlighting the beneficial effects of park density on walking rates. They report that park density is positively associated with walking for both transportation and recreation. Moreover, the presence of large trees and shaded places should be taken into account^40,44–46^. Cities often have limited space for green areas, especially in central districts. Moreover, the larger the area of green infrastructure, the more expensive land and maintenance costs become, often exceeding the budgetary means of municipalities and cities^47^. In such cases, a good solution is to create greenways—sequences of smaller and larger green spaces interconnected by pathways (incredibly pedestrian and bicycle paths—lined with vegetation where possible), elongated green areas (linear parks, boulevards along water, avenue plantings for pedestrian routs), continuing through the space, enabling longer walks, and engaging in other recreational activities.

Recent studies have also demonstrated the effectiveness of small-scale greening strategies^48^. Linear parks should be significant components of such greenway networks, mainly established along rivers, canals, and lake shores. Numerous bridges and footbridges should allow crossing to the other side of the watercourse and continuing the walk on the opposite bank. This emphasizes the importance of building a network of green spaces, or in other words, a city’s natural system, characterized by spatial continuity, hierarchical elements, and external natural connections^3^.

Our research also showed that individuals with lower stress levels more frequently exercise in green spaces, while inhabitants with high stress level rather prefer walking, seating and calm relaxation in green areas. At the same time, both, with low and high self-perceived stress, desire/need green areas. Our study suggests that water elements might be perceived as less desirable in green areas by individuals across all stress levels. However, the reasons for this trend remain unclear. One possible explanation could be safety concerns or a lower ecological awareness regarding water-related issues. Future research is needed to explore these potential factors. Previous research findings confirm, in general, the relationship between mood, quality of life, stress levels and physical activity^49,50^. At the same time, research by Barmwell et al.^51^ provides evidence that exercising outdoors has a more positive impact on stress levels than physical activity indoors.

The results of our study also showed that individuals with lower stress levels are generally more satisfied with the quality of green spaces. Furthermore, multiple regression analysis indicates that factors such as satisfaction with the cleanliness and aesthetics of green spaces, the amount of greenery, and the accessibility of green spaces contribute to reducing stress levels. Vries et al.^52^, in their research conducted regression analysis while controlling for socio-demographic characteristics. Similar to the findings of our study, they demonstrated that both the quantity and quality of street greenery were associated with stress levels. Research conducted during the pandemic, when access to green spaces was largely restricted, supports our findings. It shows that spending time in green spaces can lead to increased positive emotions and a reduction in negative feelings, such as anxiety and stress. This underscores the significance of green spaces in promoting mental well-being and highlights the importance of both accessibility and the quality of these areas in fostering social interactions and providing a sense of privacy^53–56^. However, they also noted that quality had a more significant impact on reducing stress levels than quantity, a conclusion we cannot confirm with our study results. On the other hand, a significant number of low-standard green areas causes an increase in stress levels and a decrease in perceived well-being. In this case, there is a negative relationship between the level of stress and satisfaction with greenery^57,58^.

The study by Van den Berg et al.^20^ indicates that substantial green spaces within a 3 km radius of residential areas positively reduce stress and improve overall health. Individuals with direct access to a large quantity of green space experience more significant stress mitigation related to life events than those with access to a smaller quantity of green space. In the studies conducted by Jato-Espino et al.^11^, a correlation between the location of green spaces and stress levels was also demonstrated. Difficult access to green areas and their significant distance from residential areas, as in the case of the low standard of street and urban greenery, causes frustration^59–61^. It was confirmed that the proximity of green infrastructure (GI) helped alleviate mental and behavioral health issues during the isolation conditions due to the COVID-19 pandemic; this study presented two separate case studies—the cities of La Palma and Zaragoza. Modeling for both regions was based on spatial data from the Geographic Information System (GIS) and statistical analyses; significant correlations were found between self-reported variables and greenery (GI) forms. The relationships between stress response and exposure to urban nature are also demonstrated in the research^19^. Using psychological and physiological measures employed in three studies, the correlation between exposure to urban greenery and psychological stress was assessed. In other studies, the relationships between the aesthetic values of green spaces in cities and stress recovery have been analyzed^3^, applied a method of direct assessment of a single element—the image of urban space—modeled using four parameters: the number of trees, flowers, water and small animals (birds or fish) relative to a baseline image taken in China. Aesthetic preferences for the assessed images increased with the increasing number of trees and the presence of flowers, water and fish. However, the potential for stress recovery increased mainly due to the growing number of trees, while the other elements did not have significant importance. As natural public spaces in cities, urban greenery is typically regarded not only as a place to admire the beauty of the landscape, but also for relaxation and rejuvenation after mental fatigue.

In our study, we also found that apart from elements related to the presence and quality of green spaces, stress levels are reduced by increased income and improved health status assessment, which aligns with numerous studies^62–65^. In our opinion, planning green areas in urbanized areas, policymakers and landscape planners should take under account not only preferences of residents, but also various experts’ opinions and find combined solutions.

## Limitations

While this study provides valuable insights into the relationship between perceived stress and satisfaction with green spaces, several limitations should be acknowledged.

First, although the regression model confirmed significant associations between stress levels and various greenery-related factors, it explains only a portion of the variance. This suggests that additional psychological, environmental, or socio-economic factors, which were not included in the model, may also influence perceived stress levels.

Second, despite the statistical significance of the findings, the relatively small effect sizes may limit their practical applicability in real-world contexts. While the relationships identified are meaningful, their overall impact on stress reduction may be moderate when considered alongside other life stressors.

Third, the study sample overrepresents young, educated women from urban areas. This demographic imbalance may affect the generalizability of the results, as different population groups (e.g., older adults, individuals from rural areas, or those with lower educational attainment) might experience and interact with green spaces differently. Future research should strive for a more demographically diverse sample to enhance external validity.

Lastly, while our study identifies correlations between stress and green space characteristics, it does not establish causality. Longitudinal or experimental studies would be necessary to determine the direction and nature of these relationships more definitively.

Addressing these limitations in future research will contribute to a more comprehensive understanding of how urban greenery can effectively support stress reduction across different population groups.

## Conclusions

The results suggest a significant relationship between perceived stress and satisfaction with green spaces in residential areas. Lower stress levels correlate with higher satisfaction with various aspects of surrounding greenery. Regression analysis confirms that better self-reported health status, higher satisfaction with cleanliness, aesthetics, and the quantity of greenery, as well as higher income levels, are associated with lower perceived stress. Ensuring city residents access to adequately sized, well-maintained and clean green spaces is particularly important for stress reduction.

Our study found that walking is the most common activity undertaken and evaluated highly in green spaces regardless of stress level.

Individuals with lower stress levels more frequently exercise in green spaces, while inhabitants with high stress level rather prefer walking, seating and calm relaxation in green areas. At the same time, both groups (with low and high self-perceived stress), desire / need green areas. Our study suggests that water elements might be perceived as less desirable in green areas by individuals across all stress levels. However, the underlying reasons for this tendency remain unclear. Future research could explore whether safety concerns or ecological awareness play a role in shaping these preferences.

In light of the preferences of residents experiencing stress, rather than proposing definitive solutions, our findings highlight key research fields that require further analysis and thoughtful urban planning. Therefore, such recommendations for policymakers and landscape planners should focus on ensuring access to green spaces, preferably directly, from every residential area; green spaces consisting of various elements (varying in size and richness in different forms of greenery, especially large parks); and promote diverse green space structures. Additionally, ensuring the continuity of green spaces over significant distances (linear parks, boulevards, avenue plantings along pedestrian routs) is crucial, as it allows for long walks, direct contact with greenery, and engagement in various outdoor activities, ultimately supporting residents’ well-being.

Therefore, built-up areas should be immersed in an interconnected network of green spaces (creating a system of green spaces), with greenery filling every possible niche, including greenery between and on buildings (e.g., land parcels, roofs, walls and balconies). Rather than a prescriptive approach, our study underscores the necessity of planning green networks—especially in cities, but also in other settlement units—to better integrate ecosystem services and recreational advantages into urban environments and align them with the varying needs of different social groups experiencing stress.

Such an approach will enhance ecosystem and recreational services, better addressing the needs of diverse social groups exposed to different stress levels. The research results demonstrate the importance of designing green infrastructure and planning the proper spatial relationship between built-up areas and the network of green spaces (open areas). At the same time, built-up areas should also be equipped with natural elements wherever possible. The outcome of these efforts will deliver benefits associated with improving mental health and reducing stress levels among city residents. The integration of greenery within urban environments can contribute to improve mental health and stress reduction among city residents.

## Data Availability

All data are available upon request from the corresponding author.

## References

[1] Chang, P.-J., Tsou, C.-W. & Li, Y.-S. Urban-greenway factors’ influence on older adults’ psychological well-being: A case study of Taichung, Taiwan. *Urban For. Urban Green.* **49**, 126606 (2020).

[2] Ochnik, D., Buława, B., Nagel, P., Gachowski, M. & Budziński, M. Urbanization, loneliness and mental health model—A cross-sectional network analysis with a representative sample. *Sci. Rep.* **14**, 24974 (2024). 39443642 10.1038/s41598-024-76813-z PMC11499986

[3] Wang, R., Zhao, J., Meitner, M. J., Hu, Y. & Xu, X. Characteristics of urban green spaces in relation to aesthetic preference and stress recovery. *Urban For. Urban Green.* **41**, 6–13 (2019).

[4] Grahn, P. & Stigsdotter, U. A. Landscape planning and stress. *Urban For. Urban Green.* **2**, 1–18 (2003).

[5] Coutts, C. & Hahn, M. Green infrastructure, ecosystem services, and human health. *Int. J. Environ. Res. Public Health* **12**, 9768–9798 (2015). 26295249 10.3390/ijerph120809768 PMC4555311

[6] Jimenez, M. P. et al. Associations between nature exposure and health: A review of the evidence. *Int. J. Environ. Res. Public Health* **18**, 4790 (2021). 33946197 10.3390/ijerph18094790 PMC8125471

[7] Jabbar, M., Yusoff, M. M. & Shafie, A. Assessing the role of urban green spaces for human well-being: a systematic review. *GeoJournal* **87**, 4405–4423 (2022). 34305268 10.1007/s10708-021-10474-7 PMC8290137

[8] Kaplan, S. & Talbot, J. F. *Psychological Benefits of a Wilderness Experience. Behaviour and the Natural Environment* 163–203 (Springer US, 1983).

[9] Xie, Y. et al. Credibility of the evidence on green space and human health: an overview of meta-analyses using evidence grading approaches. *EBioMedicine* **106**, 105261 (2024). 39079340 10.1016/j.ebiom.2024.105261 PMC11340586

[10] Dipeolu, A. A., Ibem, E. O., Fadamiro, J. A., Omoniyi, S. S. & Aluko, R. O. Influence of green infrastructure on residents’ self-perceived health benefits in Lagos metropolis, Nigeria. *Cities* **118**, 103378 (2021).

[11] Jato-Espino, D., Moscardó, V., Vallina Rodríguez, A. & Lázaro, E. Spatial statistical analysis of the relationship between self-reported mental health during the COVID-19 lockdown and closeness to green infrastructure. *Urban For. Urban Green.* **68**, 127457 (2022). 35002595 10.1016/j.ufug.2021.127457 PMC8717691

[12] Beyer, K. M. M., Szabo, A., Hoormann, K. & Stolley, M. Time spent outdoors, activity levels, and chronic disease among American adults. *J. Behav. Med.* **41**, 494–503 (2018). 29383535 10.1007/s10865-018-9911-1 PMC6031452

[13] Semeraro, T., Scarano, A., Buccolieri, R., Santino, A. & Aarrevaara, E. Planning of urban green spaces: An ecological perspective on human benefits. *Land* **10**, 105 (2021).

[14] Qin, J., Zhou, X., Sun, C., Leng, H. & Lian, Z. Influence of green spaces on environmental satisfaction and physiological status of urban residents. *Urban For. Urban Green.* **12**, 490–497 (2013).

[15] Franco, L. S., Shanahan, D. F. & Fuller, R. A. A review of the benefits of nature experiences: More than meets the eye. *Int. J. Environ. Res. Public Health* **14**, 864 (2017). 28763021 10.3390/ijerph14080864 PMC5580568

[16] Kumar, P. et al. The nexus between air pollution, green infrastructure and human health. *Environ. Int.* **133**, 105181 (2019). 31675531 10.1016/j.envint.2019.105181

[17] Ewert, A. & Chang, Y. Levels of nature and stress response. *Behav. Sci.* **8**, 49 (2018). 29772763 10.3390/bs8050049 PMC5981243

[18] The effects of day-to-day nature contact on workers’ stress and psychological well-being. *Urban For. Urban Green.* **66**, (2021).

[19] Kondo, M., Fluehr, J., McKeon, T. & Branas, C. Urban green space and its impact on human health. *Int. J. Environ. Res. Public Health* **15**, 445 (2018). 29510520 10.3390/ijerph15030445 PMC5876990

[20] van den Berg, A. E., Maas, J., Verheij, R. A. & Groenewegen, P. P. Green space as a buffer between stressful life events and health. *Soc. Sci. Med.* **70**, 1203–1210 (2010). 20163905 10.1016/j.socscimed.2010.01.002

[21] Beil, K. & Hanes, D. The influence of urban natural and built environments on physiological and psychological measures of stress—A pilot study. *Int. J. Environ. Res. Public Health* **10**, 1250–1267 (2013). 23531491 10.3390/ijerph10041250 PMC3709315

[22] Fleming, V. Top 10 landscape trends for 2021. *ASA Landscape Architects* https://www.asalandscapearchitects.co.uk/articles/top-10-landscape-trends-for-2021 (2021).

[23] Türksever, A. N. E. & Atalik, G. Possibilities and limitations for the measurement of the quality of life in urban areas. *Soc. Soc. Indic. Res* **53**, 163–187 (2001).

[24] Pazhuhan, M. et al. Factors underlying life quality in urban contexts: Evidence from an Industrial City (Arak, Iran). *Sustainability* **12**, 2274 (2020).

[25] Pope, D. et al. Quality of and access to green space in relation to psychological distress: Results from a population-based cross-sectional study as part of the EURO-URHIS 2 project. *Eur. J. Public Health* **28**, 35–38 (2018). 26177941 10.1093/eurpub/ckv094

[26] Cohen, S., Kamarck, T. & Mermelstein, R. A global measure of psychological stress. *J. Health Soc. Behav.* **24**, 385–396 (1983). 6668417

[27] Juczyński, Z. & Ogińska-Bulik, N. *Narzędzia Pomiaru Stresu i Radzenia Sobie Ze Stresem, Pracownia Testów Psychologicznych*. (Warszawa, 2009).

[28] Matuszczak-Świgoń, J. & Bakiera, L. Psychometric properties of the Polish version of the parental stress scale. *Roczniki Psychologiczne* **26**, 23–46 (2023).

[29] Ward Thompson, C. et al. More green space is linked to less stress in deprived communities: Evidence from salivary cortisol patterns. *Landsc. Urban Plan.* **105**, 221–229 (2012).

[30] van den Berg, M. et al. Visiting green space is associated with mental health and vitality: A cross-sectional study in four European cities. *Health Place* **38**, 8–15 (2016). 26796323 10.1016/j.healthplace.2016.01.003

[31] Park, S. H. et al. Daily park use, physical activity, and psychological stress: A study using smartphone-based ecological momentary assessment amongst a multi-ethnic Asian cohort. *Ment. Health Phys. Act.* **22**, 100440 (2022).

[32] Zhang, J. et al. Weekly green space visit duration is positively associated with favorable health outcomes in people with hypertension: Evidence from Shenzhen, China. *Environ. Res.* **212**, 113228 (2022). 35398313 10.1016/j.envres.2022.113228

[33] Vos, S. et al. Residential green space is associated with a buffering effect on stress responses during the COVID-19 pandemic in mothers of young children, a prospective study. *Environ. Res.* **208**, 112603 (2022). 34995548 10.1016/j.envres.2021.112603 PMC8730780

[34] Nielsen, T. S. & Hansen, K. B. Nearby nature and green areas encourage outdoor activitiesand decrease mental stress. *CAB Rev. Perspect. Agric. Vet. Sci. Nutr. Nat. Resour.*10.1079/pavsnnr20061059 (2007).

[35] Astell-Burt, T. & Feng, X. Association of urban green space with mental health and general health among adults in Australia. *JAMA Netw. Open* **2**, e198209 (2019). 31348510 10.1001/jamanetworkopen.2019.8209 PMC6661720

[36] Navarrete-Hernandez, P., Kiarostami, N., Yang, D. & Ozcakir, A. Green Enough? A dose-response curve of the impact of street greenery levels and types on perceived happiness. *Landsc. Urban Plan.* **251**, 105130 (2024).

[37] Wasfi, R., Steinmetz-Wood, M. & Kestens, Y. Place matters: A longitudinal analysis measuring the association between neighbourhood walkability and walking by age group and population center size in Canada. *PLoS ONE* **12**, e0189472 (2017). 29261706 10.1371/journal.pone.0189472 PMC5736224

[38] Gascon, M. *et al.* Correlates of walking for travel in seven European cities: The PASTA project. *Environ. Health Perspect.* **127**, (2019). 10.1289/EHP4603 PMC6792377 31532248

[39] Sugiyama, T. et al. Initiating and maintaining recreational walking: A longitudinal study on the influence of neighborhood green space. *Prev. Med.* **57**, 178–182 (2013). 23732245 10.1016/j.ypmed.2013.05.015

[40] Vich, G., Marquet, O. & Miralles-Guasch, C. Green streetscape and walking: Exploring active mobility patterns in dense and compact cities. *J. Transp. Health* **12**, 50–59 (2019).

[41] Zhang, X., Melbourne, S., Sarkar, C., Chiaradia, A. & Webster, C. Effects of green space on walking: Does size, shape and density matter?. *Urban Stud.* **57**, 3402–3420 (2020).

[42] Pinto, L. V., Ferreira, C. S. S. & Pereira, P. Temporal and spatial differences in human activities performed in Urban Green Spaces of Vilnius (Lithuania). *Geogr. Sustain.* **5**, 302–317 (2024).

[43] Jia, J., Zlatanova, S. & Zhang, K. Exploring spatial parameters to evaluate human walking accessibility of urban green space. *ISPRS - Int. Arch. Photogramm. Remote Sens. Spat. Inf. Sci.* **XLIV-3/W1-2020**, 73–80 (2020).

[44] Sarkar, C. et al. Exploring associations between urban green, street design and walking: Results from the Greater London boroughs. *Landsc. Urban Plan.* **143**, 112–125 (2015).

[45] Tabatabaie, S., Litt, J. S. & Muller, B. H. F. Sidewalks, trees and shade matter: A visual landscape assessment approach to understanding people’s preferences for walking. *Urban For. Urban Green.* **84**, 127931 (2023).

[46] Yu, J., Zhang, H., Dong, X. & Shen, J. The impact of street greenery on active travel: A narrative systematic review. *Front. Public Health* **12**, (2024). 10.3389/fpubh.2024.1337804 PMC10936756 38481839

[47] Meleda, P., De La Barrera, F., Contreras, C. & Reyes-Paecke, S. Berrizbeitia Cerros isla en las ciudades de Chile: oportunidades para una planificación ecológica Revista. *Revista INVI* **38**, 255–298 (2023).

[48] Tzoulas, K. et al. Promoting ecosystem and human health in urban areas using Green Infrastructure: A literature review. *Landsc. Urban Plan.* **81**, 167–178 (2007).

[49] Klaperski, S., Koch, E., Hewel, D., Schempp, A. & Müller, J. Optimizing mental health benefits of exercise: The influence of the exercise environment on acute stress levels and wellbeing. *Ment. Health Prev.* **15**, 200173 (2019).

[50] White, R. L., Ryan, D., Young, C., Elston, R. & Rossi, T. How does the context of physical activity influence perceived mood and wellbeing after exercise?. *Ment. Health Phys. Act.* **24**, 100504 (2023).

[51] Bramwell, R. C., Streetman, A. E. & Besenyi, G. M. The effect of outdoor and indoor group exercise classes on psychological stress in college students: A pilot study with randomization. *Int. J. Exerc. Sci.* **16**, 1012–1024 (2023). 37650002 10.70252/EERP4920 PMC10464750

[52] de Vries, S., van Dillen, S. M. E., Groenewegen, P. P. & Spreeuwenberg, P. Streetscape greenery and health: Stress, social cohesion and physical activity as mediators. *Soc. Sci. Med.* **94**, 26–33 (2013). 23931942 10.1016/j.socscimed.2013.06.030

[53] Szczepańska, A. & Pietrzyka, K. The COVID-19 epidemic in Poland and its influence on the quality of life of university students (young adults) in the context of restricted access to public spaces. *Z. Gesundh. Wiss.* **31**, 295–305 (2023). 33432292 10.1007/s10389-020-01456-z PMC7788176

[54] Ugolini, F., Massetti, L., Pearlmutter, D. & Sanesi, G. Usage of urban green space and related feelings of deprivation during the COVID-19 lockdown: Lessons learned from an Italian case study. *Land Use Policy* **105**, 105437 (2021). 35431392 10.1016/j.landusepol.2021.105437 PMC8996370

[55] Khalilnezhad, M. R., Ugolini, F. & Massetti, L. Attitudes and behaviors toward the use of public and private green space during the COVID-19 pandemic in Iran. *Land* **10**, 1085 (2021).

[56] Reid, C. E., Rieves, E. S. & Carlson, K. Perceptions of green space usage, abundance, and quality of green space were associated with better mental health during the COVID-19 pandemic among residents of Denver. *PLoS ONE* **17**, e0263779 (2022). 35235576 10.1371/journal.pone.0263779 PMC8890647

[57] Rigolon, A., Browning, M. H. E. M., McAnirlin, O. & Yoon, H. Green space and health equity: A systematic review on the potential of green space to reduce health disparities. *Int. J. Environ. Res. Public Health* **18**, 2563 (2021). 33806546 10.3390/ijerph18052563 PMC7967323

[58] Brumme, A. How does a “green” good affect environmental quality and social welfare?. *Jahrb. Natl. Okon. Stat.* **242**, 371–401 (2022).

[59] Moreno, C., Allam, Z., Chabaud, D., Gall, C. & Pratlong, F. Wprowadzenie do „15-minutowego miasta”: zrównoważony rozwój, odporność i tożsamość miejsca w przyszłych miastach po pandemii. *Smart Cities* **4**, 93–111 (2021).

[60] Sturm, R. & Cohen, D. Bliskość parków miejskich a zdrowie psychiczne. *J. Ment. Health Policy Econ* **17**, 19–24 (2014). 24864118 PMC4049158

[61] Cardinali, M., Beenackers, M. A., van Timmeren, A. & Pottgiesser, U. The relation between proximity to and characteristics of green spaces to physical activity and health: A multi-dimensional sensitivity analysis in four European cities. *Environ. Res.* **241**, 117605 (2024). 37956752 10.1016/j.envres.2023.117605

[62] Hamilton, H. A., Wickens, C. M., Ialomiteanu, A. R. & Mann, R. E. Debt stress, psychological distress and overall health among adults in Ontario. *J. Psychiatr. Res.* **111**, 89–95 (2019). 30690328 10.1016/j.jpsychires.2019.01.008

[63] Tsuchiya, K., Leung, C. W., Jones, A. D. & Caldwell, C. H. Multiple financial stressors and serious psychological distress among adults in the USA. *Int. J. Public Health* **65**, 335–344 (2020). 32239257 10.1007/s00038-020-01354-x PMC7274724

[64] Weissman, J., Russell, D. & Mann, J. J. Sociodemographic characteristics, financial worries and serious psychological distress in U.s. adults. *Community Ment. Health J.* **56**, 606–613 (2020). 31894440 10.1007/s10597-019-00519-0

[65] Li, R. et al. The influence of perceived stress and income on mental health in China and Germany. *Heliyon* **9**, e17344 (2023). 37408921 10.1016/j.heliyon.2023.e17344 PMC10318459

